# Carotenoid Intake and Colorectal Cancer Risk: The Multiethnic Cohort Study

**DOI:** 10.2188/jea.JE20080078

**Published:** 2009-03-19

**Authors:** Song-Yi Park, Abraham M.Y. Nomura, Suzanne P. Murphy, Lynne R. Wilkens, Brian E. Henderson, Laurence N. Kolonel

**Affiliations:** 1 Epidemiology Program, Cancer Research Center of Hawaii, University of Hawaii, Honolulu, Hawaii, USA; 2 Department of Preventive Medicine, Keck School of Medicine, University of Southern California, Los Angeles, California, USA

**Keywords:** carotenoids, colorectal neoplasms, smoking, cohort studies, multiethnic population

## Abstract

**Background:**

A protective effect of fruits and vegetables against colorectal cancer has been supported by many epidemiologic studies. This suggests that the carotenoids frequently found in these foods play a role in the prevention of this common cancer. To examine associations between the intake of individual and total carotenoids and the risk of colorectal cancer, we analyzed prospective data from the Multiethnic Cohort Study.

**Methods:**

This analysis includes 85 898 men and 105 106 women who completed a quantitative food frequency questionnaire in 1993–1996. The participants were African Americans, Native Hawaiians, Japanese Americans, Latinos, and whites aged 45–75 years at cohort entry. After an average follow-up of 8.2 years, 1292 and 1086 incident cases of colorectal cancer were identified in men and women, respectively. Cox proportional hazard models were used to estimate relative risks of colorectal cancer.

**Results:**

No significant associations were found between intake of individual and total carotenoids and colorectal cancer risk either in men or women, except for β-cryptoxanthin, which showed a mild protective effect in men. When the associations were investigated separately for colon and rectal cancer, lycopene intake was related to an increased risk of rectal cancer in men. A decreased risk was seen for total β-carotene in male current smokers, but the test for interaction with smoking status was not significant. No association was observed in each ethnic-sex group.

**Conclusion:**

Overall, our findings do not support a significant association between carotenoid intake and colorectal cancer, although some associations were seen in subgroup analyses.

## INTRODUCTION

A protective effect of fruits and vegetables against colorectal cancer has been supported by many epidemiologic studies.^1^^–^^3^ This suggests a potential role for carotenoids, which are frequently found in these foods, in the prevention of this common cancer. Several mechanisms have been proposed,^4^^–^^6^ which are related to the antioxidant properties of carotenoids in cancer prevention. Carotenoids also enhance gap junctional communication and increase immune response. Furthermore, some carotenoids, such as α- and β-carotene and β-cryptoxanthin, have provitamin A activity.

A number of studies have investigated associations between carotenoid intake and colorectal cancer risk. Results from case-control studies were inconsistent^7^^–^^17^ and recent cohort studies have reported no clear relation.^18^^–^^20^ In addition, randomized trials of β-carotene supplementation provided no evidence to support an effect of carotenoids on colorectal cancer prevention.^21^^,^^22^ The carotenoid database for U.S. foods was recently updated to include various foods^23^; however, few studies in North America have used this updated database to examine intakes of individual carotenoids putatively related to colorectal cancer.^13^^,^^15^

To further examine associations between the intake of individual and total carotenoids and the risk of colorectal cancer, we analyzed prospective data from a large multiethnic cohort study. In addition to performing overall analyses for colorectal, colon, and rectal cancer, we examined the associations according to smoking status, because cigarette smoking can alter the circulating levels of carotenoids.^24^ We also investigated the associations stratified by race/ethnicity and by multivitamin use.

## METHODS

### Study population

The design and implementation of the Multiethnic Cohort Study has been described in detail elsewhere.^25^ Briefly, the cohort consists of 215 820 men and women living in Hawaii or California who are mainly from 5 racial/ethnic groups: African Americans, Native Hawaiians, Japanese Americans, Latinos, and whites. In 1993–1996, participants aged 45–75 years completed a 26-page mailed questionnaire requesting information on dietary habits, medical history, and lifestyle practices. The study was approved by the review boards of the University of Hawaii and the University of Southern California. Voluntary return of the mailed questionnaire was regarded as an expression of informed consent on the part of the participants. For this analysis, we excluded participants who were not in one of the 5 racial/ethnic groups (*n* = 13 992) or who provided implausible dietary data (*n* = 8264). We also excluded participants with a history of colorectal cancer as reported in the baseline questionnaire (*n* = 2154) or in the cancer registries in Hawaii or California (*n* = 406). Therefore, a total of 191 004 participants (85 898 men and 105 106 women) remained for the analysis.

### Dietary assessment

Dietary intake was assessed by a quantitative food frequency questionnaire (QFFQ) that requested the frequency and amount of consumption during the past year for more than 180 food items. The QFFQ was developed from 3-day measured food records from approximately 60 men and women in each of the main ethnic groups.^25^ We performed a calibration study to assess the correlations between nutrient intakes from the QFFQ and 3 repeated 24-hour recalls for a subset of the cohort.^26^ Correlations for selected nutrients ranged from 0.57 to 0.74 for nutrient densities. For carotenoid densities, correlations were 0.60 for β-carotene, 0.54 for α-carotene, 0.51 for lycopene, 0.59 for β-cryptoxanthin, and 0.55 for lutein from foods. Daily intake of carotenoids, total energy, and other dietary components from the QFFQ were computed by applying a food composition table that has been developed and maintained at the Cancer Research Center of Hawaii for use in the Multiethnic Cohort Study,^25^ and which incorporates carotenoid values from a national database.^23^ Total carotenoid intake was calculated by summing the food intake of 5 major carotenoids: β-carotene, α-carotene, lycopene, β-cryptoxanthin, and lutein.

In addition to the QFFQ, the baseline questionnaire asked whether multivitamins or any of 7 single vitamin and mineral supplements had been used at least weekly during the previous year. If a supplement had been taken, details about the duration and frequency for that supplement were also requested. Only regular supplement use, defined as use for >1 year, was considered for this analysis. Among the 5 carotenoids examined in this study, only β-carotene as a single supplement was covered by our questionnaire. Daily supplemental β-carotene intake was calculated based on the following choices of the approximate dosage per tablet: ≤6000 µg, 7000–15 000 µg, ≥16 000 µg, and not known. β-carotene supplement users who did not provide a dosage were assigned to the lowest category (≤6000 µg). Mean daily supplement intake was calculated by multiplying the dose by the frequency of use. Carotenoid intake from multivitamin supplements was not considered because the multivitamin brands most frequently reported in the calibration study did not contain carotenoids at that time. Correlation between supplemental β-carotene intake from the baseline questionnaire and from 3 24-hour recalls was 0.43.^27^ Intake of β-carotene was reported from foods and supplements separately, and as “total β-carotene,” which was the combined intake from both foods and regularly used supplements. The consumption of other carotenoids was based on foods only.

### Identification of colorectal cancer cases

To identify incident colorectal cancer cases, the cohort was linked to the Surveillance, Epidemiology, and End Results (SEER) cancer registries covering Hawaii and California. The cohort was also linked to death certificate files in Hawaii and California and the National Death Index. Cancer identification and death information were complete through December 31, 2002. Colorectal cancer cases were limited to participants diagnosed with invasive adenocarcinoma of the large bowel. A total of 2378 incident cases (1292 men and 1086 women) were identified after an average follow-up of 8.2 years.

### Data analysis

We used Cox proportional hazards models with age as the time metric to calculate relative risks (RRs) and 95% confidence intervals (CIs) for colorectal cancer for men and women separately. Observation began at questionnaire completion or at age 45 for the few individuals who were younger than 45 at questionnaire completion, and ended at the earliest of these dates: date of colorectal cancer diagnosis, date of death, and date of closure (December 31, 2002).

We expressed daily carotenoid intakes in terms of density (intake per 1000 kcal) in the analyses, because we found that energy-adjusted intakes led to better correlations between QFFQ values and reference levels.^26^ Carotenoid intakes were divided into quintiles determined by the sex-specific intake distribution in the cohort; the lowest quintile was used as the reference group. For β-carotene models, intakes from foods (quintiles) and supplement use (yes/no) were included in the same model so that the result for each was adjusted for the other. “Basic models” were adjusted for race/ethnicity and time since cohort entry—categorized as ≤2 years, 2–5 years, and >5 years—as strata variables and age at cohort entry as a covariate. “Multivariate models” were additionally adjusted for family history of colorectal cancer, history of intestinal polyps, pack-years of cigarette smoking, body mass index (kg/m^2^), hours of vigorous activity per day, use of nonsteroidal anti-inflammatory drugs, regular multivitamin use, total energy intake (log transformed kcal/day), alcohol intake (% of kcal), red meat intake (g/day), dietary fiber intake (g/1000 kcal/day), total calcium intake (mg/day, foods and supplements), total vitamin D intake (IU/day, foods and supplements), total folate intake (mcg/day, foods and supplements), and use of hormone replacement therapy (in women only). Trend tests were conducted by including a continuous variable in the model assigned the sex and racial/ethnic-specific median values within the appropriate overall quintiles.

We conducted the analyses for colon and rectal cancer separately. Individuals with synchronous tumors at both sites (14 men and 5 women) were excluded from these analyses. We also investigated whether the association of carotenoid intakes with colorectal cancer risk differed by smoking status at baseline, by race/ethnic group, and by multivitamin use. Tests for interactions were based on the likelihood ratio test, comparing a model with main effects for the subgroup variables and the trend for the carotenoid and one that also includes interactive terms between carotenoids and subgroup membership. All analyses were performed with SAS software (version 9.1), and all *P* values were 2-sided.

## RESULTS

As compared with the entire cohort, colorectal cancer cases were older, more likely to have a family history of colorectal cancer, and less physically active, both in men and women (Table 1). In women, more cases were overweight. In men, cases were more likely to be ever smokers. For intakes of carotenoids as densities, as well as for other dietary components, there was no substantial difference between cases and the entire cohort.

**Table 1.** Baseline characteristics of cohort and colorectal cancer cases in the Multiethnic Cohort Study, 1993–2002

	Men		Women	
		
	Entire cohort (*n* = 85 898)	Cases (*n* = 1292)	Entire cohort (*n* = 105 106)	Cases (*n* = 1086)
Age at cohort entry (years)	60.2 ± 8.9^*^	64.7 ± 7.6	59.7 ± 8.8	64.2 ± 7.9
Race/ethnicity (%)				
African American	13.9	15.5	19.8	27.6
Native Hawaiian	7.0	7.0	7.4	6.2
Japanese American	29.5	38.0	27.2	30.9
Latino	24.4	19.5	21.5	15.5
White	25.3	20.0	24.1	19.9
Family history of colorectal cancer (%)	7.2	10.8	8.5	13.1
Body mass index (kg/m^2^, %)				
<25.0	43.1	44.5	49.8	45.4
25.0–29.99	42.8	42.2	30.8	31.8
≥30.0	14.2	13.4	19.4	22.8
Education (%)				
≤8 years	11.4	9.6	11.2	8.3
9–12 years	30.3	37.5	35.4	44.4
Some college or vocational school	29.1	28.8	29.5	28.5
≥college graduate	29.2	24.2	23.9	18.9
Smoking status (%)				
Never smoker	30.0	23.8	55.7	52.0
Former smoker	51.9	58.2	30.0	34.4
Current smoker	18.2	18.1	14.4	13.6
Pack-years of cigarette smoking^†^	20.6 ± 16.6	23.3 ± 17.8	15.4 ± 14.4	16.3 ± 14.6
History of intestinal polyps (%)	6.8	6.0	4.3	5.3
Physical activity (hours/day)^‡^	0.58 ± 1.02	0.44 ± 0.85	0.21 ± 0.55	0.14 ± 0.37
NSAID use (%)^§^	51.1	47.9	53.9	53.1
Ever use of hormone replacement therapy (%)			46.6	43.5
Regular multivitamin use (%)^||^	37.2	33.2	41.0	40.0
Regular β-carotene supplement use (%)^||^	4.8	4.3	4.8	4.8
Energy intake (kcal/day)	2380 ± 1105	2326 ± 1045	1947 ± 949	1855 ± 915
Alcohol intake (% of kcal)	4.0 ± 7.3	4.8 ± 8.5	1.5 ± 4.6	1.8 ± 5.9
Red meat (g/day)	72.5 ± 58.7	69.5 ± 52.0	49.3 ± 44.3	47.3 ± 40.1
Fiber intake (g/1000 kcal/day)	10.9 ± 4.2	10.5 ± 4.1	12.7 ± 4.4	12.6 ± 4.3
Total calcium intake (mg/day)^¶^	965 ± 564	888 ± 522	1059 ± 700	960 ± 647
Total vitamin D intake (IU/day)^**^	336 ± 345	298 ± 303	339 ± 347	326 ± 341
Total folate intake (mcg/day)^**^	548 ± 369	506 ± 334	528 ± 358	506 ± 357
Carotenoid intake (μg/1000 kcal/day)				
Total β-carotene^††^	2272 ± 2067	2189 ± 1786	3143 ± 2614	3194 ± 2509
β-carotene	2073 ± 1539	2047 ± 1513	2929 ± 2122	3013 ± 2138
α-carotene	449 ± 445	439 ± 429	625 ± 611	647 ± 634
Lycopene	1579 ± 1490	1550 ± 1433	1718 ± 1598	1714 ± 1815
β-cryptoxanthin	132 ± 191	136 ± 205	185 ± 261	194 ± 262
Lutein	1249 ± 1025	1223 ± 957	1653 ± 1359	1730 ± 1424
Total carotenoids^‡‡^	5483 ± 3373	5394 ± 3198	7110 ± 4323	7298 ± 4482

*Mean ± SD (all such values)

^†^For current and former smokers only

^‡^Hours of vigorous activity (vigorous work or sports) per day

^§^Nonsteroidal anti-inflammatory drugs, used at least 2 times per week for 1 month or longer during the last year

^||^Used at least once a week for more than 1 year

^¶^Intake from foods, multivitamin supplements, and calcium supplements

**Intake from foods and multivitamin supplements

^††^Intake from foods and β-carotene supplements

^‡‡^Sum of β-carotene, α-carotene, lycopene, β-cryptoxanthin, and lutein intake from foods

The basic models in men showed inverse associations between colorectal cancer risk and intake of the 5 major carotenoids, except lycopene (Table 2). However, in the multivariate models, the inverse associations were no longer significant except for β-cryptoxanthin (RR of the highest vs. the lowest quintile = 0.89, 95% CI = 0.72–1.09, *P* for trend = 0.04). Unlike in men, the basic models in women yielded no significant association except for β-cryptoxanthin intake. In multivariate models, this association was attenuated. When we ran the multivariate models without adjusting for dietary fiber intake, which was the main confounder in the association between carotenoids and colorectal cancer, a moderate inverse association was seen for β-carotene intake among men (RR = 0.77, 95% CI = 0.63–0.93, *P* for trend = 0.05, data not shown). However, the effect of β-carotene was not as strong as that of dietary fiber because the effect of dietary fiber persisted, while that of β-carotene did not, when both of them were in the model. When we examined the associations with colon and rectal cancer risk separately in the multivariate models, lycopene intake in men was significantly associated with higher risk of rectal cancer only (RR = 1.50, 95% CI = 1.04–2.16, *P* for trend = 0.018) (Table 3). No association was found between carotenoid intake and colon or rectal cancer risk in women.

**Table 2.** Relative risks (RR) and 95% confidence intervals (95% CI) of colorectal cancer according to carotenoid intake in the Multiethnic Cohort Study, 1993–2002

	Men					Women			
		
	Basic model*		Multivariate model^†^			Basic model^*^		Multivariate model^†^	
		
	Cases	RR (95% CI)	Cases	RR (95% CI)		Cases	RR (95% CI)	Cases	RR (95% CI)
Total β-carotene (μg/1000 kcal)^‡^					Total β-carotene (μg/1000 kcal)^‡^				
<1018	285	1.00	265	1.00	<1392	216	1.00	187	1.00
1018–<1468	259	0.83 (0.70–0.98)	239	0.88 (0.74–1.05)	1392–<2047	207	0.87 (0.72–1.05)	180	0.91 (0.74–1.12)
1468–<2024	241	0.72 (0.61–0.86)	215	0.79 (0.65–0.95)	2047–<12 884	202	0.79 (0.65–0.96)	159	0.78 (0.63–0.98)
2024–<3040	262	0.74 (0.63–0.88)	233	0.86 (0.71–1.05)	2884–<4355	243	0.90 (0.75–1.08)	208	1.02 (0.82–1.27)
≥3040	245	0.68 (0.57–0.81)	213	0.87 (0.70–1.08)	≥4355	218	0.79 (0.65–0.96)	186	0.98 (0.76–1.25)
*P* for trend		0.0001		0.55	*P* for trend		0.07		0.57
β-carotene (μg/1000 kcal)^§^					β-carotene (μg/1000 kcal)^§^				
<1008	289	1.00	269	1.00	<1378	220	1.00	190	1.00
1008–<1441	247	0.78 (0.66–0.93)	227	0.83 (0.69–0.99)	1378–<2012	190	0.78 (0.65–0.95)	166	0.83 (0.67–1.03)
1441–<1962	241	0.72 (0.60–0.85)	219	0.79 (0.66–0.96)	2012–<2797	217	0.84 (0.69–1.01)	173	0.85 (0.68–1.05)
1962–<2853	265	0.75 (0.63–0.88)	233	0.85 (0.70–1.03)	2797–<4126	235	0.86 (0.71–1.03)	197	0.96 (0.77–1.19)
≥2853	250	0.68 (0.57–0.81)	217	0.86 (0.69–1.06)	≥4126	224	0.80 (0.66–0.96)	194	1.01 (0.79–1.30)
*P* for trend		0.0003		0.50	*P* for trend		0.13		0.36
β-carotene supplement use^§^					β-carotene supplement use^§^				
No	1243	1.00	1121	1.00	No	1044	1.00	883	1.00
Yes	49	0.90 (0.68–1.20)	44	1.09 (0.80–1.49)	Yes	42	0.95 (0.69–1.29)	37	1.08 (0.77–1.52)
α-carotene (μg/1000 kcal)					α-carotene (μg/1000 kcal)				
<173	268	1.00	245	1.00	<225	230	1.00	196	1.00
173–<267	274	0.96 (0.81–1.14)	254	1.04 (0.87–1.25)	225–<358	187	0.77 (0.63–0.93)	161	0.82 (0.66–1.02)
267–<387	249	0.83 (0.70–0.99)	227	0.94 (0.78–1.13)	358–<543	215	0.86 (0.71–1.03)	179	0.92 (0.74–1.13)
387–<629	255	0.83 (0.70–0.99)	221	0.95 (0.79–1.16)	543–<906	226	0.86 (0.72–1.04)	186	0.96 (0.77–1.19)
≥629	246	0.76 (0.64–0.91)	218	0.99 (0.80–1.22)	≥906	228	0.85 (0.71–1.03)	198	1.07 (0.85–1.35)
*P* for trend		0.002		0.84	*P* for trend		0.55		0.12
Lycopene (μg/1000 kcal)					Lycopene (μg/1000 kcal)				
<748	290	1.00	255	1.00	<796	244	1.00	205	1.00
748–<1073	245	0.93 (0.79–1.10)	224	1.00 (0.83–1.19)	796–<1143	231	1.02 (0.85–1.22)	192	0.99 (0.81–1.21)
1073–<1435	265	1.05 (0.89–1.25)	247	1.18 (0.99–1.41)	1143–<1544	200	0.91 (0.76–1.10)	170	0.91 (0.74–1.11)
1435–<2022	251	1.04 (0.87–1.23)	221	1.12 (0.93–1.35)	1544–<2226	193	0.89 (0.74–1.08)	170	0.94 (0.76–1.15)
≥2022	241	1.00 (0.84–1.19)	218	1.16 (0.96–1.41)	≥2226	218	0.97 (0.81–1.17)	183	1.01 (0.82–1.25)
*P* for trend		0.74		0.08	*P* for trend		0.60		0.92
β-cryptoxanthin (μg/1000 kcal)					β-cryptoxanthin (μg/1000 kcal)				
<20	255	1.00	231	1.00	<30	222	1.00	188	1.00
20–<50	285	1.04 (0.88–1.23)	263	1.14 (0.96–1.37)	30–<71	206	0.88 (0.73–1.07)	185	0.97 (0.79–1.19)
50–<95	255	0.89 (0.75–1.06)	231	1.00 (0.83–1.20)	71–<134	223	0.89 (0.74–1.08)	186	0.95 (0.77–1.18)
95–<197	243	0.79 (0.67–0.95)	218	0.93 (0.77–1.14)	134–<288	204	0.77 (0.63–0.93)	169	0.84 (0.67–1.05)
≥197	254	0.72 (0.60–0.86)	222	0.89 (0.72–1.09)	≥288	231	0.80 (0.66–0.97)	192	0.94 (0.74–1.18)
*P* for trend		<0.0001		0.04	*P* for trend		0.049		0.68
Lutein (μg/1000 kcal)					Lutein (μg/1000 kcal)				
<597	264	1.00	249	1.00	<770	204	1.00	166	1.00
597–<859	266	0.95 (0.80–1.12)	234	0.93 (0.78–1.12)	770–<1114	222	1.00 (0.83–1.22)	201	1.14 (0.92–1.40)
859–<1168	255	0.85 (0.71–1.01)	222	0.87 (0.72–1.05)	1114–<1519	202	0.85 (0.70–1.04)	172	0.93 (0.74–1.16)
1168–<1682	252	0.81 (0.68–0.97)	228	0.89 (0.74–1.09)	1519–<2234	211	0.84 (0.69–1.02)	171	0.90 (0.71–1.13)
≥1682	255	0.80 (0.67–0.95)	232	0.96 (0.78–1.18)	≥2234	247	0.95 (0.78–1.15)	210	1.13 (0.89–1.43)
*P* for trend		0.01		0.98	*P* for trend		0.56		0.48
Total carotenoids (μg/1000 kcal)^||^					Total carotenoids (μg/1000 kcal)^||^				
<3093	276	1.00	257	1.00	<3912	212	1.00	180	1.00
3093–<4152	272	0.95 (0.80–1.12)	245	1.00 (0.84–1.19)	3912–<5324	219	0.96 (0.79–1.15)	190	1.01 (0.82–1.24)
4152–<5348	237	0.79 (0.66–0.93)	209	0.86 (0.71–1.04)	5324–<6958	197	0.81 (0.67–0.99)	156	0.83 (0.67–1.04)
5348–<7303	250	0.80 (0.67–0.95)	227	0.96 (0.79–1.16)	6958–<9620	228	0.89 (0.74–1.08)	198	1.04 (0.84–1.30)
≥7303	257	0.81 (0.68–0.96)	227	1.08 (0.87–1.33)	≥9620	230	0.88 (0.73–1.06)	196	1.11 (0.87–1.42)
*P* for trend		0.01		0.38	*P* for trend		0.23		0.24

*Adjusted for ethnicity and time since cohort entry as strata variables and age at cohort entry as a covariate.

^†^Adjusted for ethnicity and time since cohort entry as strata variables and the following variables as covariates: age at cohort entry, family history of colorectal cancer, history of intestinal polyps, pack-years of cigarette smoking, body mass index, hours of vigorous activity, use of nonsteroidal anti-inflammatory drugs, multivitamin use, total energy intake, alcohol intake, red meat intake, dietary fiber intake, total calcium intake (foods and supplements), total vitamin D intake (foods and supplements), total folate intake (foods and supplements), and use of hormone replacement therapy (for women only).

^‡^Intake from foods and β-carotene supplements.

^§^Intakes from foods and supplement use were included in the same model so that results for each were adjusted for the other.

^||^Sum of β-carotene, α-carotene, lycopene, β-cryptoxanthin, and lutein intake from foods.

**Table 3.** Relative risk (RR) and 95% confidence interval (95% CI) of colon and rectal cancer according to carotenoid intake in the Multiethnic Cohort Study, 1993–2002

	Men					Women			
		
	Colon cancer		Rectal cancer			Colon cancer		Rectal cancer	
		
	Cases	RR (95% CI)^*^	Cases	RR (95% CI)^*^		Cases	RR (95% CI)^*^	Cases	RR (95% CI)^*^
Total β-carotene (μg/1000 kcal)^†^					Total β-carotene (μg/1000 kcal)^†^				
<1018	188	1.00	77	1.00	<1392	146	1.00	40	1.00
1018–<1468	168	0.87 (0.70–1.08)	67	0.86 (0.62–1.20)	1392–<2047	139	0.89 (0.70–1.13)	40	0.98 (0.62–1.52)
1468–<2024	154	0.79 (0.63–0.98)	60	0.78 (0.55–1.11)	2047–<12 884	130	0.81 (0.63–1.04)	29	0.70 (0.43–1.16)
2024–<3040	167	0.86 (0.68–1.08)	64	0.86 (0.60–1.24)	2884–<4355	167	1.03 (0.80–1.31)	41	1.01 (0.63–1.63)
≥3040	157	0.88 (0.68–1.14)	50	0.76 (0.50–1.17)	≥4355	135	0.89 (0.67–1.18)	48	1.26 (0.75–2.11)
*P* for trend		0.71		0.33	*P* for trend		0.87		0.19
β-carotene (μg/1000 kcal)^c^					β-carotene (μg/1000 kcal)^c^				
<1008	190	1.00	79	1.00	<1378	148	1.00	41	1.00
1008–<1441	163	0.84 (0.68–1.04)	60	0.76 (0.54–1.06)	1378–<2012	132	0.84 (0.66–1.07)	33	0.79 (0.50–1.26)
1441–<1962	153	0.78 (0.62–0.97)	65	0.83 (0.59–1.17)	2012–<2797	135	0.84 (0.66–1.07)	38	0.90 (0.57–1.43)
1962–<2853	170	0.86 (0.69–1.08)	61	0.80 (0.56–1.16)	2797–<4126	158	0.97 (0.76–1.24)	39	0.93 (0.58–1.51)
≥2853	158	0.85 (0.66–1.10)	53	0.79 (0.52–1.20)	≥4126	144	0.95 (0.72–1.26)	47	1.19 (0.70–2.01)
*P* for trend		0.53		0.49	*P* for trend		0.73		0.27
β-carotene supplement use^‡^					β-carotene supplement use^‡^				
No	799	1.00	309	1.00	No	692	1.00	187	1.00
Yes	35	1.25 (0.88–1.78)	9	0.77 (0.39–1.52)	Yes	25	0.93 (0.62–1.41)	11	1.54 (0.82–2.91)
α-carotene (μg/1000 kcal)					α-carotene (μg/1000 kcal)				
<173	174	1.00	71	1.00	<225	152	1.00	44	1.00
173–<267	187	1.09 (0.88–1.34)	65	0.92 (0.65–1.30)	225–<358	129	0.85 (0.67–1.08)	31	0.69 (0.44–1.11)
267–<387	166	0.97 (0.78–1.21)	57	0.82 (0.57–1.18)	358–<543	144	0.96 (0.75–1.21)	34	0.76 (0.48–1.21)
387–<629	150	0.91 (0.72–1.14)	71	1.08 (0.76–1.54)	543–<906	145	0.97 (0.76–1.24)	41	0.92 (0.58–1.46)
≥629	157	0.98 (0.76–1.26)	54	0.91 (0.60–1.37)	≥906	147	1.04 (0.79–1.35)	48	1.13 (0.70–1.83)
*P* for trend		0.60		0.97	*P* for trend		0.36	44	0.18
Lycopene (μg/1000 kcal)					Lycopene (μg/1000 kcal)				
<748	189	1.00	64	1.00	<796	168	1.00	37	1.00
748–<1073	164	0.99 (0.80–1.22)	56	0.99 (0.69–1.43)	796–<1143	148	0.92 (0.74–1.15)	43	1.27 (0.82–1.98)
1073–<1435	173	1.12 (0.90–1.38)	72	1.39 (0.98–1.96)	1143–<1544	130	0.84 (0.66–1.06)	39	1.20 (0.76–1.89)
1435–<2022	159	1.09 (0.88–1.36)	60	1.23 (0.85–1.77)	1544–<2226	131	0.87 (0.69–1.10)	37	1.16 (0.73–1.85)
≥2022	149	1.06 (0.84–1.33)	66	1.50 (1.04–2.16)	≥2226	140	0.94 (0.74–1.19)	42	1.30 (0.82–2.07)
*P* for trend		0.52		0.02	*P* for trend		0.73		0.46
β-cryptoxanthin (μg/1000 kcal)					β-cryptoxanthin (μg/1000 kcal)				
<20	160	1.00	69	1.00	<30	147	1.00	41	1.00
20–<50	182	1.12 (0.90–1.39)	79	1.21 (0.87–1.68)	30–<71	142	0.95 (0.75–1.20)	42	1.01 (0.65–1.56)
50–<95	170	1.04 (0.83–1.29)	59	0.91 (0.64–1.30)	71–<134	154	1.01 (0.80–1.27)	32	0.75 (0.47–1.21)
95–<197	164	0.98 (0.78–1.23)	52	0.82 (0.56–1.20)	134–<288	126	0.80 (0.62–1.03)	41	0.93 (0.58–1.47)
≥197	158	0.88 (0.68–1.12)	59	0.88 (0.59–1.31)	≥288	148	0.93 (0.71–1.21)	42	0.92 (0.56–1.51)
*P* for trend		0.07		0.26	*P* for trend		0.61		0.91
Lutein (μg/1000 kcal)					Lutein (μg/1000 kcal)				
<597	178	1.00	70	1.00	<770	130	1.00	35	1.00
597–<859	156	0.87 (0.70–1.08)	73	1.04 (0.75–1.46)	770–<1114	161	1.14 (0.90–1.44)	40	1.16 (0.73–1.84)
859–<1168	166	0.91 (0.73–1.13)	55	0.78 (0.54–1.14)	1114–<1519	137	0.91 (0.71–1.18)	34	0.98 (0.60–1.61)
1168–<1682	166	0.91 (0.72–1.14)	60	0.86 (0.59–1.25)	1519–<2234	128	0.82 (0.63–1.07)	42	1.22 (0.75–1.99)
≥1682	168	0.95 (0.75–1.21)	60	0.96 (0.64–1.42)	≥2234	161	1.04 (0.80–1.37)	47	1.44 (0.86–2.41)
*P* for trend		0.93		0.81	*P* for trend		0.94		0.13
Total carotenoids (μg/1000 kcal)^§^					Total carotenoids (μg/1000 kcal)^§^				
<3093	180	1.00	77	1.00	<3912	143	1.00	37	1.00
3093–<4152	182	1.06 (0.86–1.31)	60	0.82 (0.58–1.16)	3912–<5324	147	0.97 (0.77–1.22)	41	1.12 (0.71–1.76)
4152–<5348	145	0.85 (0.68–1.07)	63	0.88 (0.62–1.25)	5324–<6958	123	0.81 (0.63–1.04)	33	0.92 (0.57–1.51)
5348–<7303	155	0.92 (0.73–1.16)	69	1.01 (0.71–1.45)	6958–<9620	161	1.04 (0.81–1.33)	37	1.04 (0.64–1.71)
≥7303	172	1.14 (0.89–1.46)	49	0.83 (0.55–1.27)	≥9620	143	0.99 (0.75–1.31)	50	1.52 (0.91–2.55)
*P* for trend		0.33		0.72	*P* for trend		0.74		0.10

*Adjusted for ethnicity and time since cohort entry as strata variables and the following variables as covariates: age at cohort entry, family history of colorectal cancer, history of intestinal polyps, pack-years of cigarette smoking, body mass index, hours of vigorous activity, use of nonsteroidal anti-inflammatory drugs, multivitamin use, total energy intake, alcohol intake, red meat intake, dietary fiber intake, total calcium intake (foods and supplements), total vitamin D intake (foods and supplements), total folate intake (foods and supplements), and use of hormone replacement therapy (for women only).

^†^Intake from foods and β-carotene supplements.

^‡^Intakes from foods and supplement use were included in the same model so that results for each were adjusted for the other.

^§^Sum of β-carotene, α-carotene, lycopene, β-cryptoxanthin, and lutein intake from foods.

Table 4 shows the associations between carotenoid intake and colorectal cancer risk by smoking status at baseline. There was an inverse association for total β-carotene intake in male current smokers. However, interaction between carotenoid intake and smoking status was not statistically significant in either men or women. Therefore, the associations did not differ by smoking status.

**Table 4.** Multivariate relative risks (RR) and 95% confidence intervals (95% CI) of colorectal cancer according to carotenoid intake and smoking status in the Multiethnic Cohort Study, 1993–2002

	Never smokers	Former smokers	Current smokers	*P* for interaction
	
	RR (95% CI)*	RR (95% CI)*	RR (95% CI)*	
*Men*				
No. of cases	293	664	208	
Total β-carotene^†^	0.90 (0.59–1.39)	0.93 (0.70–1.23)	0.49 (0.25–0.94)	0.20
β-carotene^‡^	0.82 (0.54–1.25)	0.93 (0.70–1.23)	0.57 (0.30–1.06)	0.48
β-carotene supplement use^‡^	1.12 (0.61–2.05)	1.12 (0.75–1.68)	0.81 (0.32–2.02)	0.87
α-carotene	0.75 (0.50–1.12)	1.11 (0.84–1.45)	0.87 (0.49–1.57)	0.63
Lycopene	1.18 (0.79–1.76)	1.14 (0.89–1.47)	1.25 (0.79–1.95)	0.75
β-cryptoxanthin	0.70 (0.46–1.08)	1.01 (0.77–1.34)	0.89 (0.53–1.49)	0.77
Lutein	0.80 (0.54–1.18)	1.11 (0.84–1.46)	0.78 (0.46–1.31)	0.41
Total carotenoids^§^	1.00 (0.66–1.52)	1.17 (0.89–1.56)	0.85 (0.49–1.48)	0.51
*Women*				
No. of cases	491	304	125	
Total β-carotene^†^	0.90 (0.64–1.26)	0.89 (0.58–1.37)	1.76 (0.89–3.50)	0.39
β-carotene^‡^	0.93 (0.67–1.31)	0.93 (0.60–1.43)	1.75 (0.87–3.51)	0.42
β-carotene supplement use^‡^	1.11 (0.70–1.77)	1.12 (0.64–1.96)	0.76 (0.24–2.48)	0.74
α-carotene	0.95 (0.69–1.30)	1.16 (0.77–1.75)	1.48 (0.80–2.70)	0.31
Lycopene	0.92 (0.69–1.24)	1.13 (0.80–1.61)	0.94 (0.52–1.69)	0.38
β-cryptoxanthin	0.95 (0.69–1.30)	0.86 (0.56–1.31)	1.00 (0.51–1.96)	0.39
Lutein	1.24 (0.90–1.72)	0.99 (0.65–1.52)	1.10 (0.59–2.03)	0.77
Total carotenoids^§^	1.00 (0.71–1.40)	1.12 (0.73–1.72)	1.64 (0.85–3.18)	0.58

*RR of the highest vs. the lowest quintile; for β-carotene supplement use, yes vs. no. Adjusted for ethnicity and time since cohort entry as strata variables and the following variables as covariates: age at cohort entry, family history of colorectal cancer, history of intestinal polyps, pack-years of cigarette smoking (for former and current smokers only), body mass index, hours of vigorous activity, use of nonsteroidal anti-inflammatory drugs, multivitamin use, total energy intake, alcohol intake, red meat intake, dietary fiber intake, total calcium intake (foods and supplements), total vitamin D intake (foods and supplements), total folate intake (foods and supplements), and use of hormone replacement therapy (in women only).

^†^Intake from foods and β-carotene supplements.

^‡^Intakes from foods and supplement use were included in the same model so that results for each were adjusted for the other.

^§^Sum of β-carotene, α-carotene, lycopene, β-cryptoxanthin, and lutein intake from foods.

In accordance with the a priori aim of the Multiethnic Cohort Study, which is to compare dietary effects across racial/ethnic groups, we investigated the associations stratified by race/ethnicity. None of the ethnic-sex groups showed statistically significant associations between carotenoid intakes and colorectal cancer risk (data not shown).

When we ran the models for multivitamin users and non-users separately, no significant association was seen in either group: for men, RR for the highest vs. the lowest quintile of total carotenoid intake = 0.94, 95% CI = 0.65–1.36, *P* for trend = 0.99 in multivitamin users and RR = 1.15, 95% CI = 0.89–1.50, *P* for trend = 0.26 in non-users; for women, RR = 1.13, 95% CI = 0.77–1.68, *P* for trend = 0.49 in users and RR = 1.10, 95% CI = 0.80–1.51, *P* for trend = 0.34 in non-users.

## DISCUSSION

In this large Multiethnic Cohort Study, we found no significant associations between intake of individual and total carotenoids and colorectal cancer risk either in men or women, except for β-cryptoxanthin, which showed a mild protective effect in men. β-cryptoxanthin has been shown to decrease colon carcinogenesis in animal models,^28^^,^^29^ and epidemiologic studies have supported a beneficial effect of β-cryptoxanthin on lung cancer,^30^^,^^31^ but not on colorectal cancer.

Prospective cohort studies have mainly reported the absence of an association. Two female cohort studies—the Canadian National Breast Screening Study (NBSS)^18^ and the Shanghai Women’s Health Study^19^—did not find significant associations between carotenoid intake and colorectal cancer risk. In male smokers in the Alpha-Tocopherol, Beta-carotene Cancer Prevention (ATBC) Study, no association was seen between dietary and serum carotenoids and colorectal cancer risk.^20^ Furthermore, a pooled analysis of 11 cohort studies also did not support an important role for carotenoid intake in the etiology of colorectal cancer.^32^ Two randomized clinical trials—the Physicians’ Health Study^21^ and the ATBC Study^22^—found no beneficial or harmful effect of supplemental β-carotene on colon/colorectal cancer. A meta-analysis^33^ of 4 randomized trials, including the ATBC Study,^34^ also reported no effect of β-carotene supplementation on primary or secondary prevention of colorectal adenoma, which is the precursor of colorectal cancer.

Findings from case-control studies have been inconsistent. Studies in Switzerland,^10^ Italy,^7^^,^^8^ China,^12^ and Japan^17^ found inverse associations of colorectal/colon/rectal cancer risk with intake of various carotenoids. However, studies in France,^14^ Canada,^15^ and Australia^16^ reported no association for intakes of different carotenoids. A study in the United Sates reported that high β-carotene intake was associated with a reduced risk for colon cancer in whites.^11^ Another study in the United States observed an inverse association between lutein intake and colon cancer risk in both men and women,^9^ and found an inverse association of lycopene intake with rectal cancer in women.^13^ In the present study, a higher lycopene intake was related to an increased risk of rectal cancer in women, which was unexpected.

Oxidative stress from tobacco smoke may modify the blood concentrations of potent antioxidants such as certain carotenoids.^24^ Only a few studies have investigated associations of carotenoid intake with colorectal cancer risk according to smoking status, and the results are inconsistent. In a large cohort of Canadian women, no association was found in never, past, or current smokers.^18^ A pooled analysis of 11 cohort studies reported that null results were not modified by smoking status.^32^ A case-control study in Canada observed that a reduced risk of colon cancer was related to high intake of β-carotene among never-smokers and to high intake of lycopene among ever-smokers.^15^ In a case-control study conducted in the United States, lutein intake was more strongly associated with decreased risk of colon cancer in current smokers than in never smokers.^9^ A case-control study in Europe found the opposite association between β-carotene intake and colorectal adenoma risk in non-smokers and in past/current smokers: a protective effect of β-carotene in non-smokers, but an adverse effect in smokers.^35^ These 3 case-control studies and the pooled analysis examined the associations in men and women combined; no sex-specific findings were reported. We found an inverse association for total β-carotene in male current smokers, but there was no interaction between smoking status and intake of carotenoids. Therefore, this finding is likely due to chance.

Although only fewer than 5% of the participants in our study reported using β-carotene supplements regularly, the dosage available in supplements is usually higher than that obtainable from foods. We might have underestimated supplemental β-carotene intake at baseline because we did not record intake from multivitamins. Some multivitamin products might have contained β-carotene, although the brands most frequently used in the calibration study did not. During the follow-up period, these common products began to contain β-carotene and other carotenoids, such as lutein and lycopene. We speculate that subsequent intake of β-carotene and other carotenoids from supplements may have been considerably higher than at baseline. However, the results for multivitamin users and non-users were similar. Lack of information on colorectal cancer screening, including colonoscopy or sigmoidoscopy findings, was also a limitation, although a history of intestinal polyps was adjusted for in the multivariate models.

The strengths of this study include its prospective design, the large number of participants, and detailed dietary information that was validated in the calibration study. A further strength was the ability to control for various factors that might potentially confound the associations between carotenoid intake and colorectal cancer. Most previous studies on carotenoid intake and colorectal cancer enrolled whites. However, we were able to examine the associations among various ethnic groups with a wide range of intakes from various sources, although we failed to find any difference between ethnic groups.

In conclusion, our findings do not support a significant association between carotenoid intake and colorectal cancer risk.
